# Coexisting tubal pregnancy and intrauterine pregnancy during a natural pregnancy, first diagnosed via ultrasonography: A case report

**DOI:** 10.1097/MD.0000000000043148

**Published:** 2025-07-04

**Authors:** Lin Pan, Shanshan Wang, Yan Huang, Ting Feng, Huaguo Shao

**Affiliations:** a Department of Ultrasound, Hangzhou Xixi Hospital Affiliated to Zhejiang Chinese Medical University, Hangzhou City, Zhejiang Province, China; b Institute of Hepatology and Epidemiology, Hangzhou Xixi Hospital Affiliated to Zhejiang Chinese Medical University, Hangzhou City, Zhejiang Province, China.

**Keywords:** heterotopic pregnancy, intrauterine pregnancy, natural pregnancy, tubal pregnancy, ultrasonography

## Abstract

**Rationale:**

The coexistence of intrauterine and extrauterine pregnancies is termed heterotopic pregnancy (HP). Most reported cases of HP occur after assisted reproductive technology, and HP in a natural pregnancy (NP) is very rare, often discovered intraoperatively. HP is a significant challenge for obstetricians, gynecologists, and sonographers.

**Patient concerns:**

A 30-year-old nulliparous woman presented to the emergency department of a local tertiary hospital on day 66 of amenorrhea with abdominal pain, nausea, and vomiting without an obvious cause.

**Diagnoses:**

Transabdominal gynecological ultrasonography revealed coexisting left ectopic pregnancy (EP) and intrauterine pregnancy (IP) in the NP, both with measurable fetal heart activity. In addition, abdominal fluid and extensive pelvic hematoma were observed.

**Interventions:**

Given a ruptured EP with hemorrhage, the obstetrician–gynecologist performed an emergency surgery, finding approximately 500 mL of blood and blood clots in the abdominal and pelvic cavities. The left tubal ampullary segment was dilated and thickened, with a visible rupture and active bleeding. Therefore, the left fallopian tube was resected, and IP was managed with therapy.

**Outcomes:**

The patient recovered well postoperatively and delivered a male infant by cesarean section at full term.

**Lessons:**

The presence of IP does not exclude the coexistence of EP, even in NP. Although HP is rare and the symptoms of EP may not be obvious, sometimes masked by IP, obstetricians, gynecologists, and sonographers must remain vigilant in this regard to reduce the rate of missed diagnoses, provide early intervention, and safeguard the health of the pregnant woman and fetus.

## 1. Introduction

The coexistence of intrauterine and extrauterine pregnancies is termed heterotopic pregnancy (HP). In ectopic pregnancy (EP), the fallopian tube is the most common site for ectopic implantation, but the cervix or abdomen may also be involved.^[1]^ EP represents a life-threatening complication during pregnancy, and early diagnosis coupled with definitive treatment is crucial to preventing maternal morbidity or mortality.^[2]^ HP in natural pregnancy (NP) is very rare, with an incidence rate of approximately 1/30,000.^[3]^ Some reports have indicated that a minority of HP can successfully result in the delivery of full-term infants, with favorable perinatal outcomes for both intrauterine and ectopic fetuses.^[4]^ However, with the increasing number of patients with infertility and the widespread use of assisted reproductive technologies (ART), such as in vitro fertilization, the incidence of HP has significantly increased.

The risk factors for HP are very similar to those for EP, including smoking, history of EP, previous pelvic inflammatory disease, sexually transmitted infections, tubal surgery, abdominal surgery, endometriosis, infertility treatments, and certain forms of contraception. Two-dimensional transvaginal sonography (TVS) is commonly used for the detection of EP; however, misdiagnosis is not uncommon. The primary treatment for HP is the resection of the EP while preserving the intrauterine pregnancy (IP). Due to the increasing use of ART, obstetricians in the future will encounter such cases more frequently, and it is also necessary to heighten vigilance towards HP, especially among patients with NP.

## 2. Case presentation

The patient is a 30-year-old woman who had never been pregnant before, with amenorrhea of over 40 days. The transabdominal sonography (TAS) conducted at our hospital indicated early IP. On day 54 of amenorrhea, the examination revealed low progesterone levels, and progesterone was taken orally once for fetus protection. On day 66 of amenorrhea, she experienced abdominal pain without apparent cause, accompanied by nausea and vomiting of gastric contents, prompting her admission to the hospital. The patient had no vaginal bleeding, fever, cough, or other discomforts. Her menstrual period was regular, with a cycle length of 29 days and a menstrual duration of 4 to 5 days, and she had no history of dysmenorrhea.

The patient had been previously healthy. She denied a history of hypertension, heart disease, diabetes, infectious diseases such as hepatitis or tuberculosis, significant drug or food allergies, long-term medication use, and follows local vaccination schedules. She denied exposure to radioactive substances or toxins and does not smoke or consume alcohol. Both parents were healthy, and the patient denied any genetic or familial diseases within 3 generations of her family, as well as a family history of cancer. The patient had an appendectomy at a local hospital 18 years ago and has not undergone any other surgical treatments.

## 3. Clinical findings

The temperature was 36.4℃, pulse was 63 beats/min, respiration was 18 breaths/min and blood Pressure was 98/50 mm Hg. The patient was conscious. A 3 cm old scar is visible on the right lower abdomen. Gynecological examination of the external genitalia revealed a married, nulliparous vagina, a smooth cervix with mild tenderness on lifting, no palpable masses, no tenderness, good mobility, thickened left adnexa with tenderness, and no significant masses or tenderness in the right adnexa.

**Figure 1.**
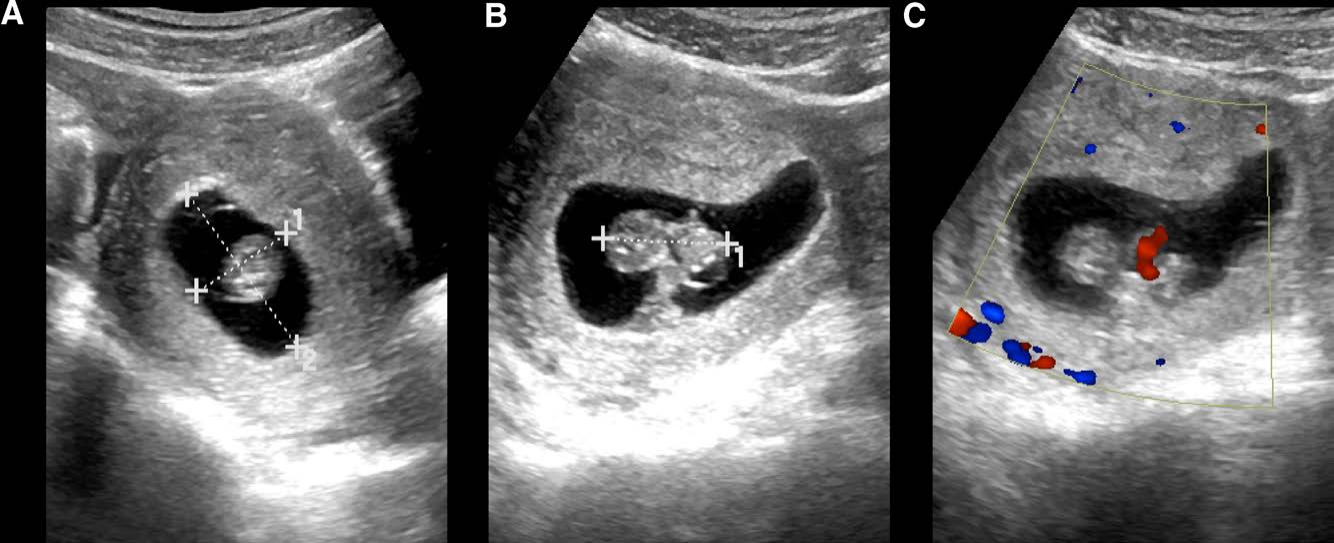
TAS results on day 66 of amenorrhea. (A) Showing IP with a gestational sac size of 55 × 24 × 42 mm. (B) Embryonic pole size of 28 mm in length. (C) CDFI revealing primitive cardiac activity within the embryonic pole. CDFI = color doppler flow imaging, TAS = transabdominal sonography.

**Figure 2.**
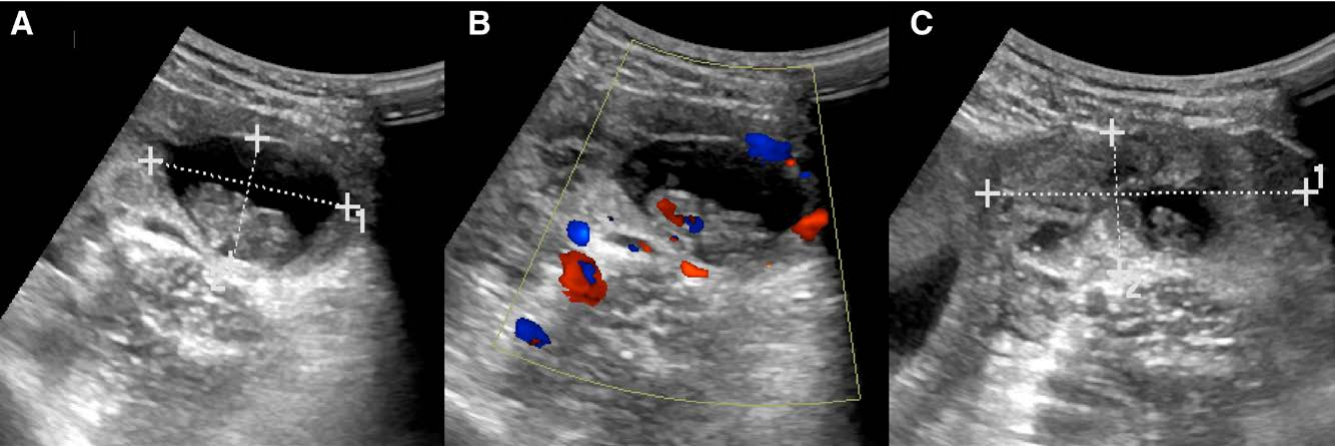
TAS results on day 66 of amenorrhea. (A) Showing EP in the left region, with a gestational sac size of 38 × 24 × 26 mm. (B) CDFI showing primitive cardiac activity in the embryonic pole of the left EP. (C) Embryonic pole size of 25 mm in length. CDFI = color doppler flow imaging, EP = ectopic pregnancy, TAS = transabdominal sonography.

**Figure 3.**
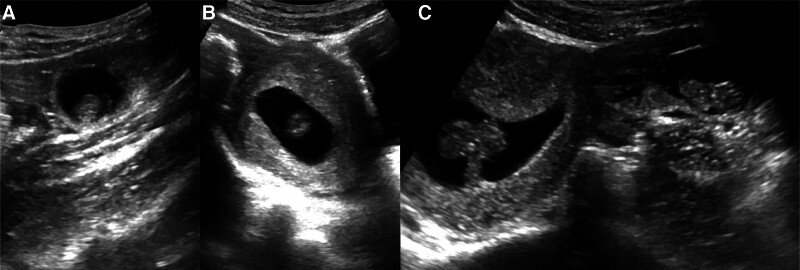
TAS results on day 66 of amenorrhea. (A) Showing HP. (B) Simultaneous showing HP on the same tangent plane. HP = heterotopic pregnancy, TAS = transabdominal sonography.

On the day of admission, laboratory tests showed a white blood cell count of 10.84 × 10^9^/L, coagulation time of 22.3 seconds, and serum hCG (Beta-human chorionic gonadotropin) level of 75,319 mIU/L. TAS revealed a gestational sac echo of approximately 55 × 24 × 42 mm in the uterine cavity, with a yolk sac and embryonic pole visible, as well as primitive cardiac tube pulsation, with a bud length of approximately 28 mm (Fig. 1). A gestational sac echo of approximately 38 × 24 × 26 mm was seen in the left adnexal area, with a yolk sac and embryonic pole visible, as well as primitive cardiac tube pulsation, with a bud length of approximately 25 mm. A hyperechoic mass of approximately 60 × 26 mm was seen adjacent to it. No significant abnormalities were found in the right adnexal area. Ascites was present in the abdominal cavity, with a maximum depth of approximately 30 mm (Fig. 2). Preliminary diagnosis were IP of a live fetus and EP of a live fetus (Fig. 3). Differential diagnosis was incomplete abortion, characterized by a history of amenorrhea, accompanied by abdominal pain or vaginal bleeding. Gynecological examination revealed a dilated cervical with retained pregnancy products or continuous blood flow, and the uterus was smaller than expected for the gestational age, which did not match the patient’s presentation. The other differential diagnosis was complete abortion, which referred to the complete expulsion of pregnancy products, with gradual cessation of vaginal bleeding and abdominal pain. Gynecological examination revealed a closed cervical and a uterus close to its normal size, which did not match the patient’s presentation.

Based on the laboratory and ultrasound findings, the patient’s current diagnosis of HP was clear. There was a possibility of ongoing active bleeding, which leaded to hemorrhagic shock and potentially life-threatening conditions if left untreated. Despite the patient’s currently stable vital signs due to compensatory mechanisms, immediate surgical intervention with salpingectomy on the affected side was indicated. The purpose of the surgery was to remove the lesion, stop the bleeding, and clear the hematoma to prevent progression to hemorrhagic shock. However, given the patient’s coexisting IP, the possibility of abortion during and after surgery could not be ruled out. The patient and her family had requested the surgery while preserving the IP, opting for an open surgical approach. They had been informed of the potential risks and complications during and after the surgery and have expressed their understanding and given informed consent. The emergency surgery, scheduled for the same day, was planned for salpingectomy on the affected side. Preoperative management included intravenous infusion of 100 mL of normal saline with 1.5 g of cefuroxime half an hour before surgery for infection prevention, and intramuscular injection 40 mg of progesterone for fetal protection.

Intraoperatively, approximately 500 mL of blood and blood clots were found in the abdominal cavity. The ampullary portion of the left fallopian tube was dilated and enlarged to approximately 60 × 40 × 30 mm, with a visible 5 × 5 mm rupture and active bleeding. The uterus was enlarged, equivalent to a 2-month pregnancy, with a smooth surface. The left ovary and right adnexa showed no significant abnormalities. Subsequently, a left salpingectomy was performed, with the placement of hemostatic agents to prevent bleeding and anti-adhesive agents to prevent adhesion. The surgery was successful, with stable blood pressure throughout. The total intraoperative blood loss and blood clots amounted to approximately 500 mL, and a total of 1500 mL of fluid was administered intraoperatively.

The patient received electrocardiographic monitoring and oxygen therapy for 12 hours. Postoperatively, 100 mL of sodium chloride solution and 1.5 g of cefuroxime was administered intravenously twice daily for 48 hours to prevent infection.

**Figure 4.**
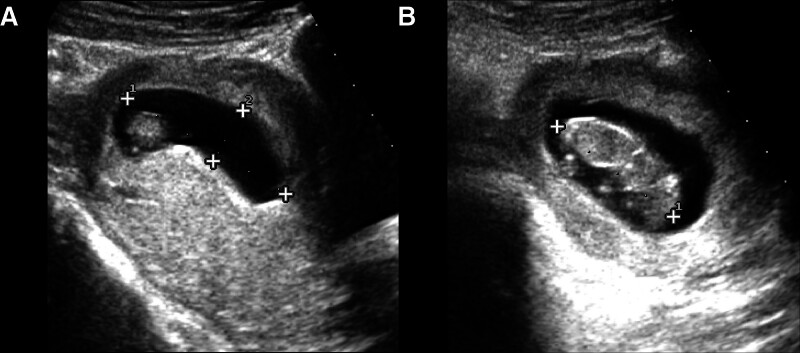
TAS results on postoperative day 8 (day 74 of amenorrhea). (A) Showing IP with a gestational sac size of 52 × 16 × 44 mm. (B) Embryonic pole size of 41 mm in length. IP = intrauterine pregnancy, TAS = transabdominal sonography.

On the first postoperative day, the patient had no chills, fever, abdominal pain, or abdominal distension. There was no significant vaginal bleeding, and the abdominal incision was well approximated with no bleeding or fluid leakage. Intravenous vitamin C was administered for fluid replacement and nutritional support. Additionally, 20 mg of progesterone was injected intramuscularly twice daily in combination with oral medicine for fetal protection. A complete blood count, C-reactive protein, and serum hCG levels were checked. On the third postoperative day, the pathology report confirmed a left tubal pregnancy. CRP was 40.90 mg/L, progesterone was 28.83 ng/mL, the red blood cell count was 3.51 × 10^12^/L, hemoglobin was 108 g/L, and the diluted β-hCG level was 54,050.0 mIU/mL. On the seventh postoperative day, progesterone was 32.25 ng/mL, estradiol was 1023 pg/mL, and the diluted β-hCG level was 52,631 mIU/mL. On the eighth postoperative day, ultrasound showed a 52 × 16 × 44 mm gestational sac with a fetus and fetal heartbeat in the uterine cavity. The head-to-rump length was approximately 41 mm, and both adnexa were normal (Fig. 4). The patient requested discharge and was discharged after consulting with the supervising physician.

During subsequent telephone follow-ups after discharge, the patient delivered a healthy male infant by cesarean section at full term in another hospital. The ultrasound examination at our hospital 17 months after delivery showed no significant abnormalities in the uterus and ovaries.

## 4. Discussion

The coexistence of intrauterine and extrauterine pregnancies is termed HP. HP is very rare in NP. In recent years, with the widespread application of ART such as ovulation induction, in vitro fertilization and embryo transfer, the incidence of HP has shown a significant upward trend, exceeding 1/100.^[5]^ The precise etiology of HP remains unclear, with numerous risk factors, including pelvic inflammatory disease, prior tubal surgery, ovarian stimulation, and ART.^[6]^ The most common site for EP is the fallopian tube, followed by the uterine cornua, while other rare locations include the ovary, cervix, and abdomen.^[7]^ Often, diagnosis is challenging due to the masking of EP by IP and the insufficient sensitivity or specificity of β-hCG levels in predicting HP. TVS is commonly used to investigate EP; however, misdiagnosis is not uncommon because many HP patients have undergone ovulation induction, and the enlarged uterus and bilateral ovaries of the IP may obscure the presence of EP.

The risk of rupture of an embryo implanted within the tubal wall is high, as abundant local vascularization and trophoblastic invasion can lead to tubal rupture, even in the absence of fetal heartbeat activity.^[8]^ β-hCG levels may continue to rise normally, and the ovaries may enlarge, but EP sacs can easily be overlooked during ultrasound scans. Despite advancements in medical knowledge, the availability of advanced ultrasound equipment, and sensitive hormone detection methods, the diagnosis of HP remains a challenge for obstetricians, gynecologists, and sonographers due to its extremely low incidence.^[9]^ The median age at diagnosis for EP is 7.5 weeks of gestation.^[10]^ EP is often asymptomatic, and it can mimic a normal pregnancy or miscarriage. In symptomatic cases, common features include vaginal bleeding, abdominal pain, signs of peritonitis, and adnexal masses.^[11]^ Some studies suggest that after confirming IP, sonographers must observe the bladder and determine the relationship between the cervix and uterus. Routine observation of the ovaries and fallopian tubes is necessary. The possibility of EP should be suspected if there are extrauterine images, suspicious fallopian tube appearance, or a significant amount of free fluid in the Douglas’ pouch^[12]^

During the patient’s first routine prenatal visit to our hospital, she had been amenorrheic for over 40 days. TAS at that time revealed an intrauterine gestational sac measuring approximately 19 × 15 × 21 mm, with a yolk sac and punctate embryonic pole visible, and a probable heartbeat. No concurrent EP was detected at that time, possibly due to the following reasons: the gestational age was relatively early, and the EP might have been too small to observe; (2) during a normal IP, especially a NP, the examining physician may subjectively overlook the possibility of an EP. Furthermore, the gestational sac in an EP often lagged in development compared to an IP, leading to a lack of observation when symptoms are inconspicuous or absent; (3) many pregnant women in China are unwilling to undergo TVS, believing it may affect the intrauterine fetus. While TAS can address most issues, it has limitations due to intestinal gas interference, which may reduce the visibility of the ovaries and other adnexal structures, potentially leading to missed diagnoses of ectopic pregnancies. When the patient was admitted to the hospital 66 days after her last menstrual period, although TAS was also performed, the EP mass was already significant, and the patient had abdominal pain. The sonographer focused on examining the location of the abdominal pain during the examination and did not overlook it due to the normal IP. Moreover, due to the rupture of the EP, there was a large amount of hemoperitoneum in the abdominal and pelvic cavities, increasing the chances of detecting the coexisting EP.

## 5. Conclusion

The presence of IP does not exclude the coexistence of EP, even in NP. Although HP is rare and the symptoms of EP may be not obvious, sometimes masked by IP, obstetricians, gynecologists, and sonographers must remain vigilant in this regard to reduce the rate of missed diagnoses, provide early intervention, and safeguard the health of the pregnant woman and fetus.

## Acknowledgments

The authors appreciate all patients and colleagues who participated in this study.

## Author contributions

**Conceptualization:** Lin Pan.

**Investigation:** Shanshan Wang, Yan Huang, Ting Feng.

**Supervision:** Lin Pan, Huaguo Shao.

**Writing – original draft:** Lin Pan, Shanshan Wang, Yan Huang, Ting Feng, Huaguo Shao.

**Writing – review & editing:** Lin Pan, Huaguo Shao.

## References

[1] Hassani KI Bouazzaoui AE Khatouf M Mazaz K. Heterotopic pregnancy: a diagnosis we should suspect more often. J Emerg Trauma Shock. 2010;3:304. 10.4103/0974-2700.66563 PMC2938513 20930992

[2] Diakosavvas M Blontzos N Daskalakis G. Vaginal delivery at term in a woman with a spontaneous heterotopic pregnancy treated with laparoscopic salpingectomy. Case Rep Obstet Gynecol. 2020;2020:8892273. 32934856 10.1155/2020/8892273 PMC7484683

[3] Bataille P Reynard A Ducarme G. Spontaneous heterotopic triplets – a review of literature. J Gynecol Obstet Hum Reprod. 2017;46:657–9. 28549987 10.1016/j.jogoh.2017.05.008

[4] Maaita ME Murad N Dabbas M. Advanced heterotopic pregnancy. J Obstet Gynaecol. 1999;19:677–8. 15512441 10.1080/01443619964120

[5] Hewlett K Howell CM. Heterotopic pregnancy: simultaneous viable and nonviable pregnancies. JAAPA. 2020;33:35–8. 10.1097/01.JAA.0000654012.56086.97 32097214

[6] Refaat B Dalton E Ledger WL. Ectopic pregnancy secondary to in vitro fertilisation-embryo transfer: pathogenic mechanisms and management strategies. Reprod Biol Endocrinol. 2015;13:30. 25884617 10.1186/s12958-015-0025-0 PMC4403912

[7] Ciebiera M Slabuszewska-Jozwiak A Zareba K Jakiel G. Heterotopic pregnancy – how easily you can go wrong in diagnosing? A case study. J Ultrason. 2018;18:355–8. 30763022 10.15557/JoU.2018.0052 PMC6444310

[8] Guimaraes AC Reis LDO Leite FC Reis CFDD Costa AP Araujo WJB. Spontaneous heterotopic triplet pregnancy with a two viable intrauterine embryos and an ectopic one with right tubal rupture. Rev Bras Ginecol Obstet. 2019;41:268–72. 30970384 10.1055/s-0039-1683910 PMC10309273

[9] Dubbewar A Srivastava A Hiremath RN Ghodke S Chourey N Sreenivas A. A rare case of spontaneous heterotopic pregnancy with intrauterine gestational trophoblastic neoplasia and tubal ectopic pregnancy at a remote secondary care hospital. J Family Med Prim Care. 2022;11:3996–8. 36387733 10.4103/jfmpc.jfmpc_2448_21 PMC9648282

[10] Soriano D Shrim A Seidman DS Goldenberg M Mashiach S Oelsner G. Diagnosis and treatment of heterotopic pregnancy compared with ectopic pregnancy. J Am Assoc Gynecol Laparosc. 2002;9:352–8. 12101334 10.1016/s1074-3804(05)60416-1

[11] Waheed H Masroor I Afzal S. Quadruplet heterotopic pregnancy; ectopic managed successfully with laparotomy with subsequent viable intrauterine pregnancy: a case report. Radiol Case Rep. 2022;17:1528–31. 35273675 10.1016/j.radcr.2022.02.007 PMC8904177

[12] Cimpoca B Moldoveanu A Gica N. Heterotopic quadruplet pregnancy. literature review and case report. Medicina (Kaunas). 2021;57:483. 34065925 10.3390/medicina57050483 PMC8151375

